# Challenges and Solution Directions for the Integration of Smart Information Systems in the Agri-Food Sector

**DOI:** 10.3390/s25082362

**Published:** 2025-04-08

**Authors:** Emmanuel Ahoa, Ayalew Kassahun, Cor Verdouw, Bedir Tekinerdogan

**Affiliations:** 1 Information Technology Group, Wageningen University and Research, Hollandseweg 1, 6706 KN Wageningen, The Netherlands; emmanuel.ahoa@wur.nl (E.A.); ayalew.kassahun@wur.nl (A.K.); bedir.tekinerdogan@wur.nl (B.T.); 2 Wageningen Social and Economic Research, Wageningen University and Research, Droevendaalsesteeg 4, 6708 PB Wageningen, The Netherlands

**Keywords:** smart farming, data interoperability, smart systems, ontology, semantic interoperability, system of systems

## Abstract

Traditional farming has evolved from standalone computing systems to smart farming, driven by advancements in digitalization. This has led to the proliferation of diverse information systems (IS), such as IoT and sensor systems, decision support systems, and farm management information systems (FMISs). These systems often operate in isolation, limiting their overall impact. The integration of IS into connected smart systems is widely addressed as a key driver to tackle these issues. However, it is a complex, multi-faceted issue that is not easily achievable. Previous studies have offered valuable insights, but they often focus on specific cases, such as individual IS and certain integration aspects, lacking a comprehensive overview of various integration dimensions. This systematic review of 74 scientific papers on IS integration addresses this gap by providing an overview of the digital technologies involved, integration levels and types, barriers hindering integration, and available approaches to overcoming these challenges. The findings indicate that integration primarily relies on a point-to-point approach, followed by cloud-based integration. Enterprise service bus, hub-and-spoke, and semantic web approaches are mentioned less frequently but are gaining interest. The study identifies and discusses 27 integration challenges into three main areas: organizational, technological, and data governance-related challenges. Technologies such as blockchain, data spaces, AI, edge computing and microservices, and service-oriented architecture methods are addressed as solutions for data governance and interoperability issues. The insights from the study can help enhance interoperability, leading to data-driven smart farming that increases food production, mitigates climate change, and optimizes resource usage.

## 1. Introduction

The agri-food sector, encompassing food production, logistics, and processing, accounts for a substantial portion of the economy, particularly in developing countries. Traditionally lagging behind in adopting digital technologies, the sector is currently digitalizing at a rapid pace [1,2,3,4]. Since its start in the 1980s and 1990s, digitalization in agriculture has evolved from single computer and limited information sources to more advanced systems [5,6,7,8]. These advancements in digitalization have led to and enabled smart farming [9,10,11]. Initially mainly reliant on satellite data, smart farming now is integrating advanced technologies such as the Internet of Things (IoT), big data analytics, quantum IoT, AI-driven digital twins, artificial intelligence (AI), and digital twins [12,13,14].

Although the transition from single computing systems to smart farming has significantly enhanced farming operations, it has also led to a proliferation of diverse information systems (IS) that hardly communicate [15,16,17,18,19,20]. These systems, including IoT sensing and control systems, farm management information systems (FMISs), geographic information systems (GIS), supply chain systems, and decision support systems, are often used in isolation, operating independently [21,22]. This fragmentation limits the overall impact of these technologies by making it challenging to combine systems from different sources [23,24,25]. For example, inadequate data interoperability constrains the development of smart systems such as Machine Learning (ML) and generative AI, which rely on large datasets from diverse sources [26,27,28].

The integration of information systems (IS) into fully connected systems is widely addressed as a fundamental challenge to tackle these issues within the agri-food sector [3,29,30,31]. Integration not only facilitates technical interoperability but also unlocks benefits that go far beyond what the standalone systems can offer [29,32]. Advanced precision farming applications, for example, are enabled by the integration of field, crop, and climate sensors with AI-based decision support systems and farm equipment control [33,34,35]. Similarly, climate and other sensors can be integrated with greenhouse control systems to automatically optimize plant growth [36,37]. Moreover, the studies of [38,39,40] demonstrated that integrating blockchain technology with IoT and AI can significantly enhance transparency and food traceability. Additionally, the large datasets from such unified systems have also been recognized for their potential in the development and deployment of artificial intelligence models for decision making. In terms of information sharing, IS integration could enhance the freshness of information (Age of Information) by optimizing (near) real-time updates through seamless collaboration among the systems [41,42,43]. Up-to-date data from IoT sensors can trigger automated technologies to adjust resource usage, thereby optimizing resource utilization and facilitating timely decision making in smart farming [44,45].

However, realizing such a full integration is a complex, multi-faceted issue that is not easily achievable [25,46,47,48]. Data interoperability remains a significant hurdle, as incompatible data formats, procedures, and protocols are deeply embedded in existing systems [49,50]. Moreover, data security concerns must be addressed to establish trust [51,52]. Achieving effective integration requires collaboration and coordination among partners, but conflicting interests can hinder this [53]. Finally, successful integration demands approaches that consider not only the technological aspects but also sector-specific challenges, such as business process heterogeneity, varying technological literacy, and diverse partners [54,55,56].

Although existing studies [17,52] have provided valuable insights into the aforementioned integration dimensions, they are often limited to specific application areas or technologies, such as FMISs, blockchain, data spaces, IoT, and AI [19,20,51,53]. Consequently, the findings from these studies are scattered across various sources, causing a lack of a comprehensive overview of the different aspects of integration in smart farming. This study seeks to address the gap by conducting a systematic literature review (SLR) to thoroughly analyze the current state of IS integration and to explore future prospects towards a fully integrated smart farming system.

The main aim of this research is to provide valuable insights into the challenges and potential solutions for integrating diverse digital technologies to advance future smart agriculture. Specifically, the study will provide an overview of the current IS landscape, existing types of integration, barriers that hinder integration, and available approaches for mitigating these barriers. To achieve this objective, the following research questions (RQs) were formulated:

- RQ1: What are the information systems discussed in the literature studies?
- RQ2: What are the levels of information system integration in the agri-food sector?
- RQ3: What are the different types of integration reported in the agri-food sector?
- RQ4: What are the current approaches for achieving information system integration?
- RQ5: What are the challenges hindering information system integration and potential solutions?

The remainder of this paper is organized as follows: Section 2 discusses the methodology employed in the paper. Section 3 presents the results of the SLR. The discussion of the study and conclusion are presented in Section 4 and Section 5, respectively.

## 2. Research Methodology

**Figure 1:**
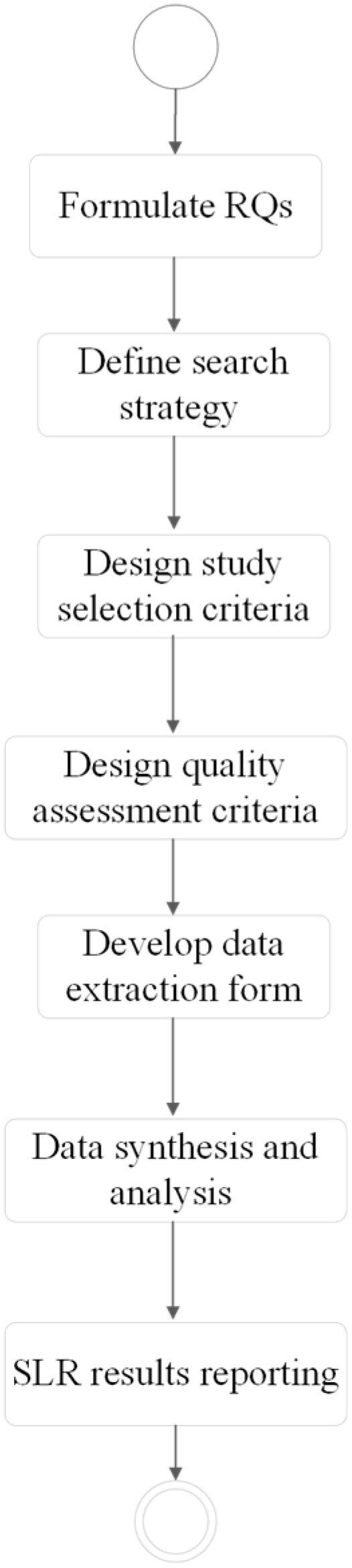
Activity diagram showing SLR process (adapted from [57]).

In this study, we used the systematic literature review (SLR) protocol of [57] that follows the steps shown in Figure 1. Firstly, the research question (RQ) was formulated based on the main objective of the study. From the formulated RQ, we defined our search strategy (step two), which was used to search potential studies. As part of the research protocol, we selected the database, constructed search strings, and performed the search using the defined strings. The search strings were tested on selected databases and improved iteratively to meet the requirements of the databases and to find as many relevant studies as possible. Finally, the resulting search strings were used to query the studies (step three).

Next, the results were selected, and an assessment was performed to determine the relevance of the studies using the study selection criteria (step four) and subsequently the quality assessment technique. In step five, we created an extraction form in Microsoft Excel (Appendix A) to collect and retrieve relevant information from the selected articles. In the sixth and seventh steps, which are the last steps in the methodology activities, we synthesized the data and presented the results derived from the extracted data.

### 2.1. Search Strategy

We developed a query string and searched publications in Scopus and Web of Science, which indexed nearly all peer-reviewed scientific publications on our research topic. We adopted both automated and manual search processes. This search protocol helps to broaden the search results and has also been applied in related studies [58,59,60].

To formulate the search string for the automated search, we first identify the key elements or concepts in the review question, which include integration of information systems and agri-food. Because of the differences in terms of requirements related to the database, the search string was adapted to meet the criteria of the database.

The syntax presented in this paragraph shows the query for Scopus, which was altered slightly for Web of Science, but the key concept and search terms remained the same for both databases. The search string was pre-tested with the selected database and the string was improved accordingly before the final search was implemented. The final search string used for the SLR is in the syntax form: (((“systems of system*” OR “business systems integration” OR “information integration” OR “data sharing” OR “interoperab*” OR “data integration” OR “application integration” OR “enterprise system* integration”) AND (“floricultur*” OR “horticultur*” OR “agri-food” OR “agro-food” OR “agrifood” OR “agrofood” OR “value chain*” OR “agricultur*”))).

**Figure 2:**
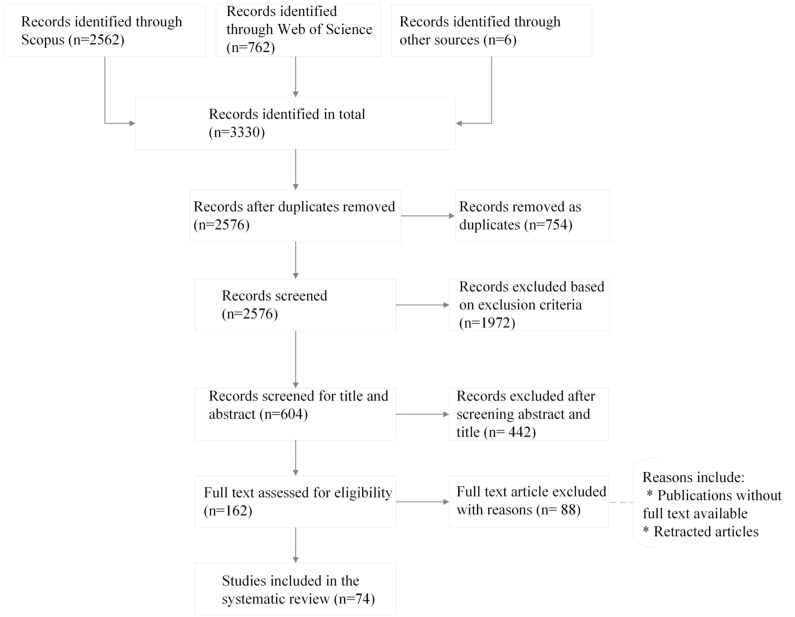
Overview of search result and study selection procedure.

Finally, the search for the articles was performed on 20 January 2025 and this yielded a total result of 3330 articles for the automated search (Figure 2). Most of the studies were found in Scopus with search string results of 2562, and 762 were found in Web of Science. After the duplicates were removed, we applied a manual search in the process of snowballing (backward/forward) to search the reference list of the studies obtained from the automated search. This additional step was carried out to ensure no records were inadvertently overlooked. Six additional articles that met the inclusion criteria of the study were found from cited references search, i.e., records identified through other sources.

### 2.2. Study Selection Criteria Strategy

To select the relevant studies to help fulfil the study’s objective, we formulated seven inclusion criteria (IC) (Table 1). These criteria were applied on the large number of potential journals, conference papers, and book chapters retrieved from the different database to retain the appropriate papers. Papers that check-mark all the formulated selection criteria in Table 1 were material for the review study. Papers that do not meet all the criteria were tagged as less relevant and excluded. The studies excluded are as follows: (a) technical report, working papers, literature reviews, books, unpublished theses, project deliverables, retracted papers, and poster publications; (b) studies that do not fall within the time frame 2010 to 2024; (c) overlapping papers between Scopus and Web of Science, one was retained and the other excluded; (d) publications that do not validate our review aim; (e) non-English related articles and (f) publications with scope not relating to the agri-food sector, publications focusing on livestock/animals were excluded. All the criteria were developed in an iterative process with all the authors involved in the study.

In applying IC 4 (Table 1) to the 3330 articles, 754 studies were removed as duplicates. We observed that the majority of records obtained from the Web of Science search results appeared as duplicates in the Scopus results. We screened the remaining records of 2576 further with exclusion criteria (a)–(e). The outcome of this activity resulted in 604 papers which were screened for title and abstract. The significant decrease in articles was primarily due to limiting publications to our study’s subject areas, focusing specifically on IS integration in the agri-food sector. Excluding systematic literature review papers also played a significant role in reducing the number of articles. The titles and abstracts of the 604 papers were read in Microsoft Excel, after performing the IC 5 and IC 6, 442 studies were excluded. There were 162 papers that qualified for the full text assessment. The full text of the 162 papers were read thoroughly and the selection criteria (IC 5, IC 6, and IC 7) were performed. After applying the selection criteria, 74 papers were selected to be material for the primary study (Figure 2). Then, 88 full text articles were excluded. Some of the publications were excluded with reasons such as full text not available (Table 1).

### 2.3. Quality Assessment

Before extracting and synthesizing the primary data, we assessed the quality of the 74 papers during the reading of the full text. The criteria (Table 2) for assessing the quality of the data were based on the quality instrument, as highlighted by [61] and the studies of [62,63]. The goal of the quality assessment is to ensure that high quality material is included in the primary studies.

**Figure 3:**
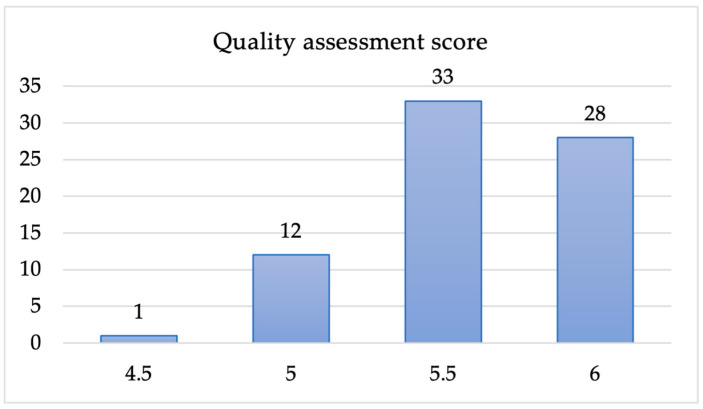
Quality score distribution of 74 primary studies.

We assessed each of the 74 papers using the criteria (Table 2) and graded them on a three-point quality scale (1 = when the criteria are fully met, 0.5 = when the criteria are partly met, and 0 = when the criteria are not met). For example, if the aim of the study (Q1 from Table 2) is clearly stated, a score of 1 is awarded, and no point is awarded if the study aim is not stated. A score of 0.5 is awarded if the study aim is not clearly or vaguely stated. The aim was to exclude studies with a score lower than 3 points from the primary study list to ensure the quality of the study is met. After the rating of each paper with the quality scale, we noticed that each of the 74 studies made a score higher than the minimum score of 3. Because of this, all 74 papers we included in the primary studies to proceed to the data extraction stage. The outcome of the quality assessment is presented in Figure 3.

### 2.4. Data Extraction

To systematically extract the relevant information from each of the selected studies, we first developed a data extraction form (Appendix A) in Microsoft Excel. The data extraction form contains columns such as current IS, level of IS integration, types of integration, and approaches and challenges of integrating IS. Second, we conducted preliminary testing of the form using a sample of studies from the Scopus database. The pre-test was carried out to avoid inconsistencies, and to also check validity and reliability of the form. Third, the form was adjusted to eliminate ambiguities and incompleteness that were discovered during the piloting phase. In the earlier version, we discovered that we were missing a column to extract the solution directions for the integration challenges. Finally, the full text of each study was read, and the data were extracted and documented in the final version of the data extraction form.

### 2.5. Data Synthesis and Analysis

The information gathered were then synthesized into tables and charts to give a clear presentation of the study results. In a situation where there are synonyms, an umbrella concept was used to harmonize and categorize such cases (e.g., the list of current IS, challenges hindering IS integration and approaches for IS integration). The current IS, approaches for integrating IS, and challenges of integrating IS were identified qualitatively and directly from the primary studies. Feature modelling, an approach in software product line engineering for visualizing feature abstraction and relationships, was used to present the integration dimensions (Section 3.1). The feature model presents features in a hierarchical tree-like structure, with additional levels of cross-tree relationships [64,65].

### 2.6. Threats to Validity and Mitigating Strategies

In this section, the probable threats to validity including the corresponding mitigating strategy are highlighted. First, the study employed systematic review protocol as outlined in the study of [57] to ensure all the steps required to achieve the goal of the study are followed. Secondly, looking at the possibility that the study could suffer from construct validity and may not measure what we expect to measure. Because of this, we followed a step-by-step approach to define the search string. Also, for each concept, we define the search terms (synonyms, related terms, and spellings). We ensure that the synonyms, related terms, and spellings are based on related studies in the literature. This was supported with the *, “AND” and “OR” Boolean operators. The search queries were reviewed and tested multiple times among the researchers before being used for the final search. Following this approach, there could be a chance that some key studies were missed due to the exclusion criteria; however, looking at the final list of 74 papers, we believe this is a reasonable amount for the SLR study.

Also, the likely threat of screening and selection bias was addressed with predefined inclusion and exclusion criteria. These criteria were reviewed thoroughly by all the researchers to ensure such a threat is minimized. During the screening, we noticed that some papers, e.g., [55,66,67], even though they have high quality and could contribute to achieve the research goal, were still excluded as they do not meet the inclusion criteria. We also developed a data extraction form to record the information extracted from the primary studies. Because the extraction form plays a key role in the research and could influence the results, the data extraction form was reviewed among the researchers and pre-tested multiple times. Based on the feedback from the authors and lessons learned from the pre-test, the form was updated before using it for the final data extraction.

During data synthesis, we ensured that the content analysis approach was adopted to classify the data. To address the validity with the classification of the data, all the terms and themes used for the classification were based on the existing literature related to the data identified from the primary study.

Publication bias, which often occur in a situation where researchers only publish the positive outcome of a study rather than the negative outcome, was stated to be part of the key threats to validity [61]. To mitigate this threat, we developed a list of a quality assessment criteria which we used to assess the quality of each of the 74 papers selected for the primary study. This approach helped to rate each paper to ensure the quality of the SLR study is met; the goal was to only include papers with a score higher than the minimum score of 3. We also ensured that the primary goal of the research was aligned to the data synthesis to contribute to answering the research question.

## 3. Results

### 3.1. Feature Model for Information System Integration

**Figure 4:**
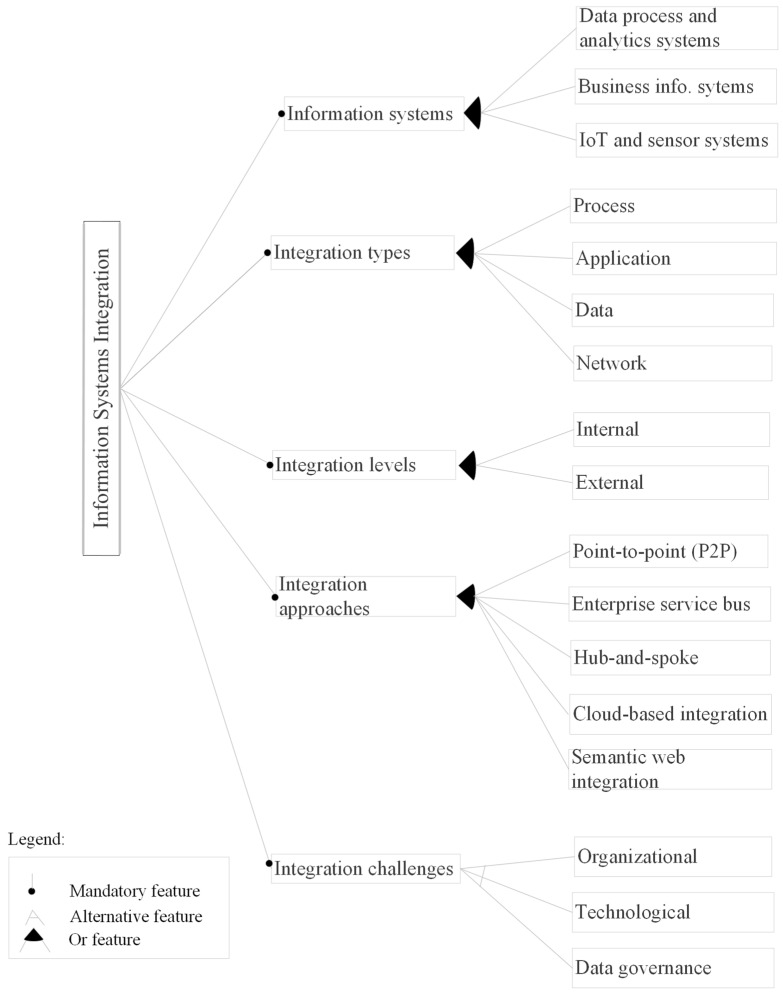
Feature model showing dimensions of IS integration in agri-food sector.

The feature model in Figure 4 presents the thematic areas from the literature analysis. Each of the five themes represent key aspect of IS integration for supporting fully integrated smart farming. This was also used to organize the findings from the literature review presented in this section (Section 3.1, Section 3.2, Section 3.3, Section 3.4, Section 3.5, Section 3.6 and Section 3.7), with each theme corresponding to each research question and result.

- Information systems: This dimension examines the IS used in the agri-food sector from the literature review (Section 3.3). The study also delves into the functionalities provided by these diverse IS. IoT and sensor systems were the most addressed IS in the reviewed papers (more than 50%).
- Integration levels: This integration aspect assesses the hierarchical level at which IS integration could occur (Section 3.4). The integration levels were grouped into internal integration (integration within the agri-food organization) and external integration (integration between at least two independent organization). Although other integration levels were identified, the majority of the studies highlighted external integration.
- Integration types: This section of the integration dimensions focuses on the different ways in which the process, application, data, and network work together in a unified whole (Section 3.5). Most papers focus on integrating the vast amount of data in the agri-food sector. Out of the 74 papers analyzed, data integration had the most occurrences (69), appearing in 70 papers.
- Integration approaches: This dimension analyzes the available methods used to integrate IS. It explores various approaches (Section 3.6) that can be employed to integrate the agri-food processes, applications, data, and network. From the 74 studies reviewed, 62 explicitly mentioned one or more of such approaches.
- Integration challenges: Integrating heterogeneous processes, applications, data, and networks to exchange data and coordinate processes presents challenges relevant to the integration process. In this part of the integration aspects, we pinpoint these obstacles that hinder the integration of IS and possible solutions. From the 74 papers analyzed, 27 distinct challenges were identified, encompassing both technical and non-technical issues. These challenges were derived from 46 of the papers (Section 3.7).

### 3.2. Overview of Selected Studies

**Figure 5:**
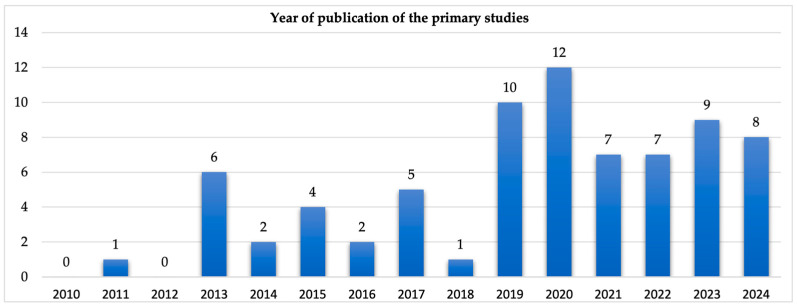
Number of articles published per year.

Figure 5 shows the distribution of the number of the primary studies published between 2010 and 2024. We observed that most of the studies (30%) were published in 2019 and 2020. The relatively large number of papers in 2019 and 2020 compared to the number of papers published between 2010 and 2018 indicate that the integration of data and systems in the agri-food sector became prominent in early 2019. No paper was found in 2010 and 2012, with only one paper published in 2011. This resulting data imply that though integration of IS could be popular in other sectors during those periods, however, in the agri-food sector, it was still maturing. In Appendix B, we present a list of the 74 primary studies used in this research.

**Figure 6:**
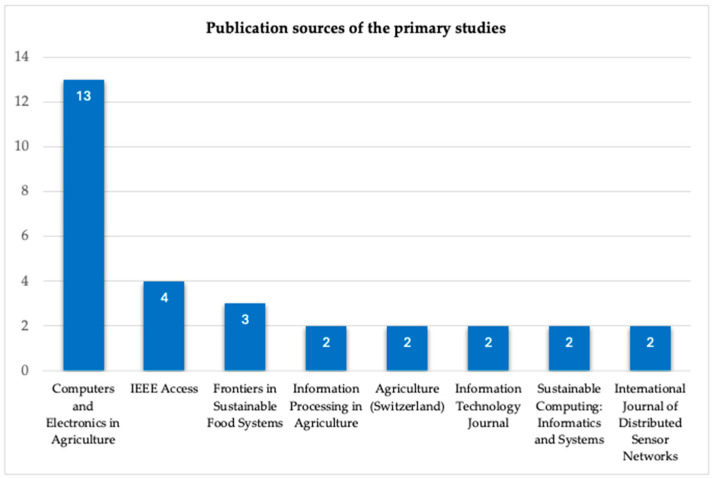
Top eight publication sources.

Concerning the publication avenues, we observed that the 74 primary studies were published in 52 different journals. Figure 6 shows the eight journals where more than one paper was published. Moreover, 30 of the papers were published in the top eight journals together, with the remaining 44 published in 44 different journals. *Computers and Electronics in Agriculture* and *IEEE Access* seem to be the most preferred journals among researchers in the agri-food and IS integration field.

**Figure 7:**
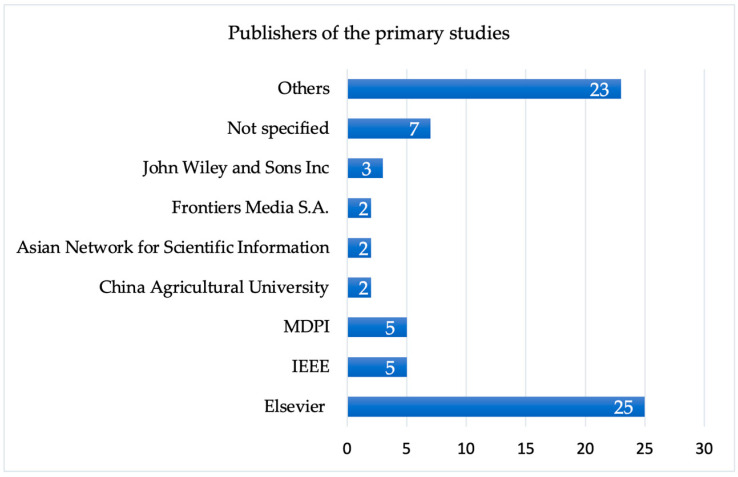
Number of papers per publisher.

In Figure 7, we present the number of papers published by the different publishers. Out of the 74 articles, the majority, i.e., 25 papers, were published by Elsevier. This is followed by the Institute of Electrical and Electronics Engineers Inc. (IEEE) and MDPI, which have each published five papers. The data analysis results show that 23 of the papers were each published by different publishers (labelled as Others in Figure 7) whilst for seven of the papers, the names of the publisher were not specified (Figure 7).

**Figure 8:**
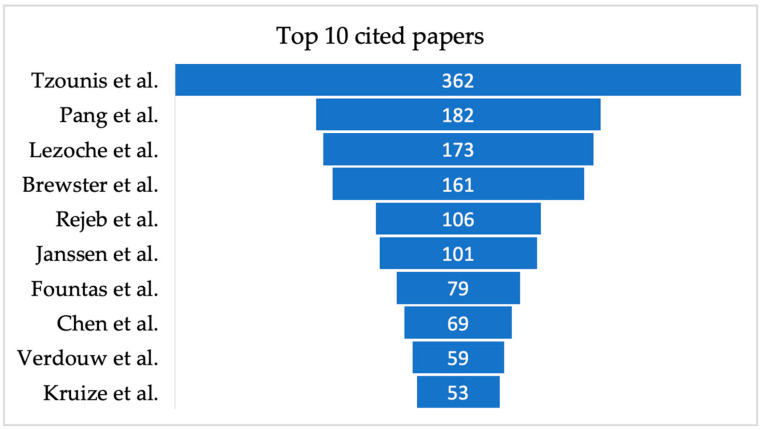
Top ten most cited papers.

In addition to the above general statistics, the top ten most cited papers are shown in Figure 8. The study of Tzounis et al., which focused on advances and future challenges of integrating IoT such as RFID and other sensors, was observed to be the most cited paper with 362 citations. This was followed by Pang et al.’s publication (182 citations), which proposes a business–IT joint framework for integrating information to create value. Lezoche et al., which surveyed latest digital technologies as well as the integration process across the technologies, was the third most cited paper (173 citations). These papers were followed by studies conducted by Brewster et al., Rejeb et al., and Janssen et al., which have 161, 106, and 101 citations, respectively. The remaining four most cited papers, Fountas et al. and Chen et al., recorded 79 and 69 citations, respectively, while the papers of Verdouw et al. (59 citations) and Kruize et al. had 53 citations (Figure 8).

**Figure 9:**
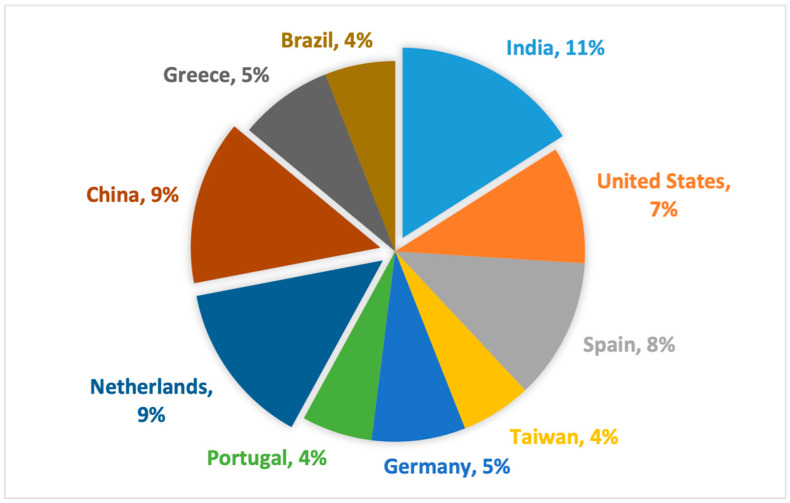
The top ten countries that contributed three or more papers to the studies.

Lastly, we analyzed the geographical distribution of the studies. Figure 9 presents a pie chart illustrating the top ten major contributors to IS integration in the agri-food sector. India, China, and the Netherlands stand out as the leading contributors, with 8 (11%) of the 74 papers originating from India. China and the Netherlands each produced seven studies (9%), followed by Spain and the United States with six (8%) and five (7%) studies, respectively. Germany and Greece contributed four (5%) studies each, while Brazil, Portugal, and Taiwan are in tenth place with three (4%) studies each.

### 3.3. Information Systems in the Agri-Food Sector

From the analysis results, we noticed that 70 out of the 74 studies mentioned one or more IS used by the stakeholders in the agri-food sector to support the business processes. Though we observed repetitions and similarities in the list of the identified technologies, the individual IS includes AI-based technologies, IoT such as sensors, cyber security (e.g., preserving systems), unmanned aerial vehicles (UAVs), GIS, blockchain, robotics, and communication technologies (e.g., smartphones). Business information systems such as ERP, FMIS, and supply chain systems were also addressed in the reviewed papers. Other systems such as monitoring applications like cameras, notification systems [28,48], and semantic technologies [68] were also mentioned.

Based on their functions, applications, and data management capabilities, we categorized the extracted technologies into (1) Data processing and analytics systems, (2) Business information systems, (3) IoT and sensor systems, and (4) Other systems. Table 3 presents these categorizations and the specific technologies.

- *Data processing and analytics systems*: All the technologies with the capabilities to manage data and information and providing decision support were listed under this category (Table 3). Data processing and analytics systems was the second most occurred technologies (about 30%) from the primary studies. The IS in this category uses the knowledge of human and existing data to make predictions, alerts, and accurate measurements to make the agri-food processes efficient and predictable [69,70]. In the studies of [28,71,72], AI models, IoT devices, and blockchain applications were used to make crop and climate predictions, control product quality, improve transparency, and decentralize transactions in the agri-food sector. Recently data spaces are introduced as a federated approach for sharing data while maintaining data sovereignty [18,48]. Ontology and semantic technologies were seen to be part of the promising technologies in the agri-food sector. They are used for creating knowledge and facilitating data interoperability, respectively. Moreover, two [73,74] of the 74 primary studies highlight the usage of ontology systems in the form of ontology look up services in the agri-food sector. The study of [73] reveals that such technologies contain a list of datasets and vocabulary that are often stored on the web using W3C Web Ontology Language (OWL). This can then be used to search for a known dataset.
- *Business information systems:* The systems in this category (Table 3) are “all in one” applications, which help to integrate the business functions that include production, quality management, logistics and warehouse management, supply chain management, customer relationship, purchasing, sales, and accounting. Business IS provide software solutions for the storing and retrieval of data and information. IS from this category were extracted from studies including [3,16,18,75]. FMISs dominated the list of the business IS extracted from the studies with 16 times occurrences followed by enterprise resource planning system (ERP), which was listed by four of the literature studies [48,76,77,78]. FMIS is recognized as the main software for farm operation management, planning, reporting, and record keeping. It utilizes a structured database to provide farmers with set of functionalities for storing and organizing farm data [3,74].
- *IoT and sensor systems:* These digital technologies are characterized with their data collection, connectivity, remote monitoring, and integration capabilities. They use sensors, satellite imagery, robotics, and monitoring cameras to support shop floor and monitor environmental conditions. IoT and sensor systems were the most occurred digital technologies from the primary studies. More than 50% of the primary studies, e.g., [79,80,81,82,83], mentioned at least one or more adoption of such systems, making it one of the most widely used technology in the agri-food sector. IoT and sensor systems’ prospective roles for supporting data interoperability in the agri-food sector were predominant among the recent studies [69,84,85]. To analyze data generated from precision farming technologies, IoT was used to build decentralized and interoperable infrastructure for executing the training and inference stages of deep learning algorithms [69].
- *Other systems:* Infrastructure and cyber security, cloud computing, and digital twins, which serve as backbone for the available technologies, were also identified [68,86,87,88]. Cyber security systems provide a supporting role to ensure data security, privacy, and integrity in the agri-food sector [86].

### 3.4. Level of IS Integration

From the literature, two main levels of IS integration were identified. Out of the 74 studies, 58 of the papers mentioned at least one or more scope for integrating technologies. Sixteen of the studies such as [79,87,89,90,91,92] did not indicate the level of integration. About 41 of the studies highlighted external integration. Within external integration, the integration mostly happens across the supply chain level, e.g., in the studies of [82,93,94,95]. Other external integration scopes such as regional, global, industry, and integration involving government processes and IS were revealed [3,29,96]. In the study of [3], the separate IS and processes of farmers, contractors, rural professionals, and consumers were considered in the IS integration. The 17 studies that centre on internal integration primarily focuses on farm, e.g., [74,97,98,99,100,101], and field levels, e.g., [102,103,104]. Within internal integration, for one of the studies [101], the integration occurred within a greenhouse.

### 3.5. Types of Information System Integration

**Figure 10:**
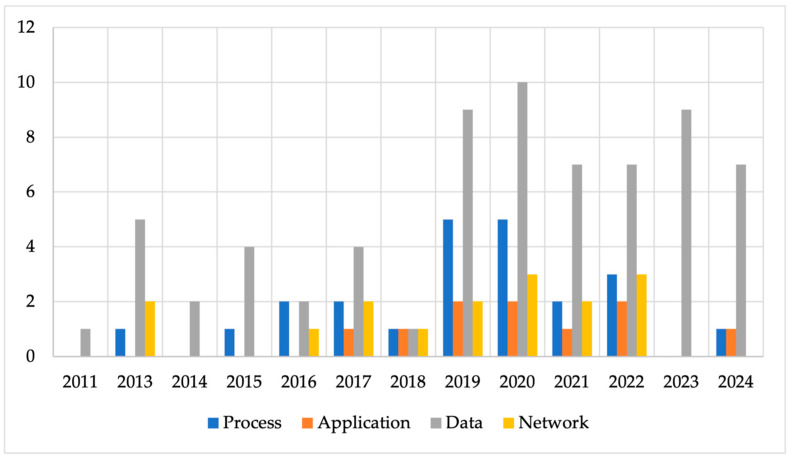
Occurrences of integration types addressed by analyzed papers (see references in Appendix C).

The selected studies were also assessed to identify the different layers at which integration occurs in the agri-food sector. The main integration types such as process, application, data, and network were identified either singly or combined in 70 of the 74 publications from 2011 to 2024. Another type such as domain integration was identified [71]. Four studies [87,89,90,91] did not specify the integration type for the technologies mentioned. In most articles, the focus of the integration is data. Figure 10 shows that data integration occurred frequently in almost all the papers from 2011 to 2024, except for one [92]. This is followed by process and network integration, each accounting for 33% and 23% of the 70 articles, respectively. Application integration was the least discussed topic in the analyzed articles, accounting for only 14% of the 70 papers.

Data integration being the most frequently mentioned element underscores its fundamental role in IS. Data integration is defined as the process of retrieving and collating different types of data from many sources into a standardized format for easy access and sharing [28,48,68,73,105,106,107,108,109]. Though there are many categories of data and information that could be merged, the study results reveal examples of such data to include climate and weather, farm and field operations, sensor information, historical data, and supply chain information [78,100,109]. This is further illustrated in the study of [3], where data from various sources, including third-party software systems, FMIS, data analytics platforms, hardware, and user-facing web-based applications, were integrated. One study [106] asserts that some analysis techniques such as predictive and prescriptive could be applied to the integrated data to extract knowledge to optimize yield, obtain insights into environmental changes and reduce costs and pesticides resistance.

In addition to data integration, the majority of the studies (33%) proposed process integration as part of the integration type that could support the overall integration of IS. This was mostly seen among studies published from 2015 to 2022. These papers constitute 30% of the total 33% from the 70 articles. The process integration level involves the alignment of the tasks, activities, and processes performed within and outside the enterprise to ensure seamless operations. Process integration is crucial as it is this integration type that bridges the technologies and the business components [27,78,110]. It allows for process automation and the creation of technological solutions through the usage of business services. The results of the reviewed articles indicated that process integration goes beyond aligning the coordinating activities and their interactions; however, it also extends to the integration of the resources (e.g., human) with the technology and data to fully optimize the workflow [28,48,83,94,111,112]. Finally, in the reviewed study of [83], process integration was sub-divided into information management, operations execution, production control, and physical object based on the ISA-95 integration reference model. In a typical agri-food supply chain, the information management layer involves the integration of processes on an aggregate level related to the control of the entire enterprise (e.g., farm, field, etc.). On the other hand, operation processes are related to the planning and control of the input materials through to the end products. Production control specifies the integration of the tasks performed by the human work force and equipment. The physical object process visualizes the integration of the physical objects which include plants, logistics boxes and containers, farm equipment, and trucks on farm, field, and supply chain levels.

Some papers (14%) mention application integration as part of the integration types for integrating systems to fully harness their functionalities. Unlike process integration, application integration tends to be the focus of publications between 2017 and 2022. A total of 9 articles out of the 10 publications from the 70 studies were published during this time frame. This integration type involves integrating two or more systems to simultaneously work together to facilitate seamless storage, management, and flow of data and information [101,112,113,114,115]. The study of [115] expatiated further on application integration with an example in the context of the Internet of Things. In the authors’ view, application integration does not only facilitate the integration of the different technologies but also provides middleware platforms for handling heterogenous data [16]. Additionally, important services such as the storage of data, data access, and data analytics, which help provide support farmers decision making, can be derived through application integration [18,27]. An example could be the gathering of IoT data which often come from sensing devices such as sensors, data directly recorded by humans, and commercial data generated from business processes. These different sources of data could be sent through a gateway to an intermediary platform, which is the IoT platform, where the data are stored and occasionally processed before sending the information to the user-facing devices. Finally, per the analyzed results, the integration of applications in the agri-food sector is predominantly driven by IoT-based technologies, with data processing systems such as blockchain, data spaces, and AI emerging as the second most utilized technology. This conclusion is supported by the fact that, among the 10 analyzed studies addressing application integration, 40% exclusively focused on IoT-based technologies. This also explains the dominance of IoT and sensor technologies among the digital technologies in the agri-food sector.

Concerning network integration, we observed that it was also not often mentioned in the primary studies. The lower count implies that IS integration in the agri-food sector focuses more on data and process integration than just network communications. However, network integration is prominent among papers published between 2016 and 2022. In nine of the papers where all the four main integration types (data, process, application, and network) are mentioned, network integration is cited as crucial in IS integration [28,48,71,75,83,97,116,117,118]. This type of integration serves as the medium that provides the transport and communication of data throughout the applications. The analysis results from the studies of [72,96] provided an example of network integration in IoT technologies. Such network integration could include the integration of different kinds of wired and wireless devices with farm equipment such as weeding machine, harvester, and tractor for transporting and communicating data with machines, sensors, and cloud management support systems.

### 3.6. Approaches for Integrating Information Systems

In Section 3.5, we presented the identified integration types from the literature. The results for achieving these integrations are presented in this section. Twelve unique approaches, point-to-point (P2P), cloud-based integration, service-oriented architecture (SOA), blockchain, data standards GS1, domain-specific language (DSL), microservices, multi-agents, hub-and-spoke, enterprise service bus (ESB), and the use of semantic and emerging technologies (AI, ML, etc.), were identified.

**Figure 11:**
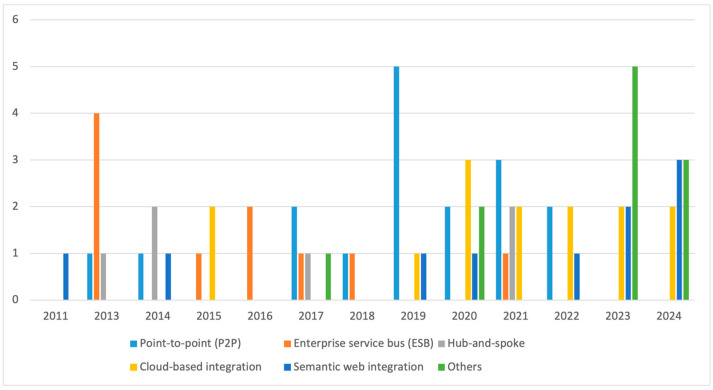
Overview of integration approaches per year (occurrences of integration approaches).

In 62 of the reviewed studies, one or more approaches for integrating systems were clearly mentioned. The remaining papers (12) did not specify any approach for achieving integration. Some approaches for, e.g., point-to-point (P2P) and cloud-based integration, occurred repetitively. The extracted approaches were grouped into (1) point-to-point (P2P), (2) enterprise service bus (ESB), (3) hub-and-spoke, (4) cloud-based, and (5) semantic web integration (Figure 11). Other integration approaches such as service-oriented architecture (SOA), cloud computing, microservices and domain-specific language (DSL) were also addressed. Moreover, 17 out of the 62 publications proposed the use of point-to-point for integrating IS, making it the most frequently mentioned method. Following point-to-point is the use of cloud-based integration, which was also present among 14 of the publications (Figure 11). Both enterprise service bus and semantic web integration occurred 10 times each. Hub-and-spoke was the least frequently used, occurring only six times. Appendix D provides an overview of the integration approaches and respective studies.

Figure 11 shows that the integration of IS in the agri-food sector is only emerging recently. Before 2017, only a few integration approaches were addressed. In 2011, 2015, and 2016, for instance, semantic web, ESB, and cloud-based were the only approaches discussed by the analyzed studies. This can be a result of less usage of these technologies in these periods as integration of systems often occurs when there is an increase in the application landscape of an enterprise. A similar explanation that might also hold for the few integration approaches in these periods could be attributed to the fact that system integration, even though it was prevalent in other sectors, was still at its infant phase in the agri-food sector. Semantic web methods, though initially mentioned in papers published in 2011, 2014, 2019, and 2020, have shown a notable increase in their application, particularly in recent publications (2022, 2023, and 2024). This could be attributed to their novelty in the agri-food sector.

In terms of their use cases, the study of [104] demonstrated the P2P method where smart services were built into a knowledge-based system of systems (SoS). Using an application programming interface (API) as the point-to-point communication protocol, different technologies, ontology systems, mobile applications, drones, satellites, and field machines, were directly connected to each other, enabling them to share data.

Unlike the P2P approach which has no intermediary, ESB was observed to use a specialized intermediate system called service bus that serves as a common service layer connecting the different systems. The service bus is perceived as a middleware and message bus that links the different systems together. In the studies of [68,97,110], ESB was used as a middleware for extracting, converting, and standardizing data from different sources. To achieve this, different services (e.g., service data) were designed together with the service bus. These services when called execute the corresponding functional block to synchronize the flow of data across the systems. As an additional layer, an enterprise data bus was added to provide access to data from other enterprise application systems or databases. This approach was frequently found in studies published between 2015 and 2016. These studies contributed 4 articles to the overall 10 studies from the 62 articles.

Cloud-based integration utilizes cloud platforms to integrate various IS. For integrating AI with IoT in smart farming, the study of [69] demonstrated the uses of the cloud-based integration method for deploying AI algorithms to analyze data from IoT systems. Also, to analyze data from various sources in the agri-food sector, [119] used cloud computing to develop a platform providing data integration, analysis, and visualization services. In a separate study, [18] proposed the cloud-based integration approach for enabling data interoperability and addressing data sovereignty. The results showed that the cloud-based method is often combined with approaches like P2P, ESB, and point-to-point [108]. Out of the 10 articles that discuss this approach, 6 of them were published in recent years (2022 to 2024).

With the hub-and-spoke approach, all the connections between the different applications are handled by a message-oriented broker labelled as the hub. The hub, which serves as the central engine of the integration receives, translates and directs messages to their destinations. A customized form of this approach (AgDataBox API) using a specialized web application was applied in precision farming [105] to store and integrate different data formats. For this specific use case, the communication between the server and other applications happens with an API. The API receives and stores the data in an organized format which is later transferred to the different client applications when a query search is executed. The hub-and-spoke approach first appeared in an article published in 2013 [106] and was last mentioned in 2021 in the study of [99]. The advancements in emerging technologies like blockchain, robotics, sensors, AI, and ML are driving the need for a flexible and scalable integration approach, which might be the reason behind the shift from the traditional hub-and-spoke model to cloud-based approaches.

For a semantic web approach, formal semantics are used to give meaning to data gathered from disparate systems. While the previous approaches rely on communication protocols, semantic integration uses vocabularies like AGROVOC [73], ontologies, and linked data to facilitate data interoperability [68,107,108]. First, a language is developed to describe the interrelations of data in a format that can be read and processed by other machines. The data are represented in a form of a graph using standards such as Resource Description Framework (RDF) [87,95,107] and Web Ontology Language (OWL). These standards are then used to produce semantic annotation models. The annotations can then be applied on pre-processed data with many formats and from different sources. This helps to produce semantic data that can be used to describe data elements and their relationships with other data represented within the same or other datasets to form a knowledge graph. Data stored and managed in a semantic web can be queried using the semantic query language (SPARQL). This querying language can be used to query different data from different databases and systems. Following these steps [85], developed agri-food-specific ontology for IoT interoperability. For enhancing agri-food supply chain data sharing, [75] employed web semantic to integrate decision support systems, FMISs, and mobile apps. There was a rise in publications focusing on this approach, similar to cloud-based approaches, between 2022 and 2024. Among the eight papers published in 2024 that explore integration approaches for IS, three papers [27,75,95] focus on semantic integration.

In addition to the above integration approaches, other methods including service-oriented architecture (SOA) [16,81], microservices [120], and multi-agent system [101] were derived from the primary studies. These approaches involve structuring software components as a service so they can be (re)used to integrate different systems. To enable seamless sharing of data and knowledge among farmers in the European agri-food sector, service-oriented architecture (SOA) was used in the study [81] to integrate various heterogenous IS (FMIS, IoT) and hardware. A hybrid approach involving emerging technologies to fully integrate digital technologies for data sharing was noticed in the literature studies [29,84,121]. Using this approach, cloud platforms, AI, blockchain, a decision support system, and cloud and edge computing were integrated to enable the seamless integration of IS in smart farming. Separate functions were allocated for the technologies, including the cloud platform for data collection and AI for data integration and analysis. The blockchain, decision support system, and cloud and edge computing systems provide data security and governance services. From the 10 articles that discuss such approach, eight of them were published in 2023 and 2024.

### 3.7. Challenges Hindering IS Integration and Potential Solutions

**Figure 12:**
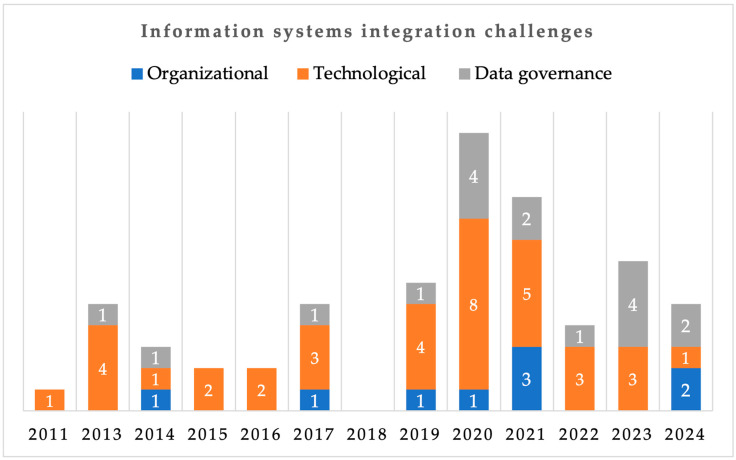
Distribution of integration challenges addressed over years (occurrences per challenge).

A list of challenges and barriers for achieving IS integration in the agri-food sector was identified from 46 of the primary studies (Table 4). For the remaining 28 studies, integration types and approaches were sometimes mentioned, but none addressed integration issues. In total, 27 unique challenges were identified, and these were grouped into (1) Organizational, (2) Technological, and (3) Data governance. Each of the 46 articles mentioned one or more of these categories. Three papers [78,100,118] discussed all the integration categories. Figure 12 illustrates the 63 occurrences of these challenges over the years (2011–2024), while Table 4 presents the specific challenges for each categorization.

The limitation for achieving IS integration were less addressed by the papers published between 2011 and 2018 (Figure 12). This perception was mostly limited to non-technical integration challenges (organizational and data governance). Technological issues, on the other hand, occurred 13 times out of the 18 occurrences in this period (Figure 12). This confirms our earlier results in Section 3.6 on the limited integration approaches between these periods, as the challenges are often apparent during IS integration. From 2019 to 2021, an increase in occurrences were noticed for organizational challenges. A similar result was derived for the data governance challenges in 2019 and 2020. This suggests that challenges in integrating IS in the agri-food sector shifted to consider non-technical barriers during this period. Papers published in 2023 and 2024 emphasized data governance issues in the integration of IS. This trend could be attributed to factors such as trust and transparency in data sharing, data privacy, and ethical and legal considerations.

- Out of the 63 occurrences (Figure 12), organizational issues accounted for 14%. From the nine occurrences of organizational issues, the majority (4) were seen among articles published between 2019 and 2021. The authors in [3,122] discussed agri-food stakeholders’ willingness to share resources and effectively collaborate as challenging to achieve IS integration. It can be deduced from [3,48] that organizational challenges are largely dependent on the level to which the processes, stakeholders’ roles, and responsibilities in the sector are aligned towards a common goal and objective to make services more accessible [3,48]. This often resulted from a mismatch between the information needs [48,118], inadequate skilled resources, and a lack of clarity of organizations’ business models [78,118]. Achieving IS integration requires decentralizing systems from partner organizations; however, the IS in the current agri-food sector are centralized and internally focused so integration beyond basic sharing of data is challenging [3]. To contribute to addressing organizational challenges, the authors in [3,28] proposed mapping the individual organizations to an overall integrated landscape. This includes the use of a commonly agreed modelling method to align, integrate, and document the business processes and data flows.
- Technological challenges: In the integration of IS, technological issues were seen to be more prevalent than data governance and organizational issues. They accounted for 59% of the 63 occurrences, making it the most pressing issue for integrating IS. The majority of the technological issues were identified through studies published between 2019 and 2024. Notably, 2020 articles had the highest number of occurrences, with a total of 8. The challenges in this category were reported to come from poor communication infrastructure, vendor and in-house IS heterogeneity, and inadequate data distribution services [99,100,123,124,125]. Quality attribute issues such as latency and throughput, scalability, reliability, and data processing power were also identified [72,75,86]. Furthermore, data interoperability issues reported to come from the independent IS were discussed frequently among the analyzed studies [16,18,81,120]. Data interoperability issues were observed to arise from heterogenous data types and formats from the separate technologies identified in Section 3.3. Similarly, the authors in [86,96,117,118] attributed the data interoperability issue to the different underlying protocols of the IS. To address the technological issues especially data interoperability, the authors in [16,28,112,126] proposed developing common standards and vocabularies that are understandable by all stakeholders and the use of standard language such as agroXML, which describes agri-food production processes and real-world objects. The use of semantic technologies approaches such as Resource Description Framework (RDF) and Web Ontology Language (OWL) could also help to tackle the data integration issue [73,107,108]. Other solutions such as the use of microservice and SOA to enhance data processing and analysis, providing flexibility and scalability among the different hardware and software components, were identified [3,120,122].
- Data governance challenges: Integration issues related to data governance accounted for 27% of the integration challenge occurrences, making it the second most significant issue, besides technological challenges. These issues primarily stem from the absence of standardized protocols, laws, and regulations governing the integration and exchange of data within the agri-food sector [18,87,120]. Integrating IS across organizations increases the risk of data privacy and security breaches, hindering data sharing and information utilization [72,75,78,91,100]. Disputes regarding data ownership and usage rights can arise especially when dealing with diversified stakeholders, IS, and dynamic sectors like agri-food (Table 4). Data governance challenges can be addressed by adopting blockchain technologies, which brings about transparency in distributed ledgers [121,127] and data spaces that enable federated data sharing and data sovereignty [18,48]. Furthermore, the use of multi-instance platform architecture could also help address privacy and security issues. Such platforms add a security layer such as confidentiality and anonymity between the implemented applications and the network layer [122].

**Table 4:** Identified challenges to be aware of when integrating IS.

Category	Specific Challenges	No. of Papers	Study
Organizational	Diversity of organizational data and systems	9	[3,48,71,78,100,118,121,122,128]
	Unclear business models		
	Mismatch between information needs of stakeholders		
	Dynamics and complexity of the sector including variety of business processes		
	Inadequate skilled resources		
Technological	Siloed data applications and systems	37	[3,16,18,28,68,72,73,74,75,76,77,78,80,86,88,91,92,94,96,98,99,100,101,102,103,107,109,114,117,118,120,123,124,125,128,129,130,131]
	Lack of alignment of data and systems		
	Network problems		
	Heterogenous in vendor and in-house systems		
	Lack of alignment of system architectures		
	Poor communication infrastructure		
	Incompatible network specifications		
	Complexity of systems		
	Underlying stack challenges		
	Inadequate data distribution services		
	Lack of flexibility in the software components		
	Poor data integrity		
	Poor scalability		
	Data processing power		
	Reliability of data and system issue		
	Latency and throughput issue		
	Magnitude/volume of data		
Data governance	Data security	17	[28,72,73,86,91,96,99,100,114,115,117,118,123,124,125,128,132]
	Lack of standardization		
	Data ownership		
	Data accessibility issues		
	Business privacy issues		

In addition to the above results, we saw that most of the technological challenges such as network problems, poor communication infrastructure, and siloed data applications and systems could be perceived when integrating data, applications, and networks using any of the integration approaches. Moreover, organizational issues such as inadequate skilled resources are often visible when using modern approaches such as semantic web technologies to integrate data and applications. Also, we observed that integrating data and systems using semantic web technology approaches require technical know-how and such knowledge is scarce in the agri-food sector [107]. Data governance challenges especially data security and privacy are also obvious when integrating data and application with any of the approaches identified from the literature review.

## 4. Discussion

The main contribution of this review lies in its systematic analysis of the fundamental aspects of integration required for smart farming systems. To do so, a thematic framework outlining the key dimensions of IS integration was developed. While this framework was specifically applied to identify critical aspects of IS integration within the agri-food domain, it can also be valuable for similar studies in other sectors.

In our review of 74 papers (published between 2010 and 2024), most papers (30%) were published in 2019 and 2020, with only one paper in 2011 and 2018. No paper was published in 2010 or 2012. This result demonstrates the infancy of the concept of IS integration in the agri-food sector between 2010 and 2012. A similar explanation that could also hold is that the agri-food sector had not fully adopted smart farming technologies during this period. Although existing studies [133,134] published before 2010 discussed and acknowledged smart farming technologies and potentials, the research of [133] also noted that the widespread adoption and implementation of smart farming was not advancing during those periods. Most agri-food organizations and farms relied on single computing systems, which reduced the need for integrated IS. Our results further substantiate this observation, as we found that most integration approaches were published between 2013 and 2024.

Likewise, many of the studies were inclined to digital technologies and integration but do not include the organizational and digital maturity levels in integrating the IS. Well-established interoperability maturity models, such as the model of ISO 11354 [135,136] or the LISI maturity model [137,138], are very valuable for extending the analysis in future research. Furthermore, the framework of this study, as presented in the feature model, was derived from the results of the literature review. It aligns well with these models and other established interoperability frameworks, though future research could address this relation more explicitly.

The results from the geographical distribution reveal India, China, and the Netherlands as the main contributors to the global IS integration in the agri-food sector. This aligns with a similar study [60] where India and China were also identified as part of the major contributors to data analytics platforms for agriculture system development. The limited number of studies from developing regions such as Africa could mean that studies from such economies appear to focus on digital technology adoption instead of IS integration. Future research could contribute to address this imbalance by focusing on both digital technology adoption and IS integration as the current review focuses more on digital technology integration and with less emphasis on adoption. This will help shed light on technology adoption in relation to IS integration dimensions. 

For the first research question, we analyzed the digital technologies that were addressed in the reviewed papers. A wide variety of over 40 distinct IS were identified, which seems to confirm the broad awareness and the increasing use of digital technology in the agri-food sector. A similar conclusion was reached by [66,67,139,140]. The large variety of systems addressed also demonstrates that these technologies serve different purposes. For instance, the technologies in the IoT and sensor systems category, which was the most prevalent in the studies, provide real-time data collection and monitoring to improve efficiency and make daily decisions. Other data processing and analytics IS include AI, semantic web, blockchain, data space, and technologies such as cyber security, cloud computing, and digital twins are becoming increasingly recognized in the sector. This affirms the statements of [34,45,140] who concluded that the agri-food sector is moving from only collecting and storing data to a data-driven sector.

Additionally, the study found that beyond data processing and analytics capabilities, existing technologies such as semantic systems could help facilitate and enable interoperability of diverse IS. This dual role of data processing technologies aligns with previous studies [141,142,143] where the role of advanced technologies such as AI in addressing data integration issues are demonstrated. However, the role and impact of AI technologies, such as Machine Learning (ML), deep learning (DL), generative AI, blockchain, and IoT systems combined with IS integration were explicitly not discussed in the reviewed papers. Similarly, despite the role played by cyber security systems in addressing food supply chain security issues, such systems were not commonly mentioned by the 74 papers. The potential reason for these limitations could be due to the slow adoption of digital technologies in the sector.

To address the second research question, the levels of IS integration were identified and examined. The review showed that the scope of integration is diverse, encompassing both internal integration and external integration that can occur at regional, global, industry, and government levels. This result goes beyond previous studies [144,145,146] which identified internal and external integration as the only scope for integrating digital technologies. In the current literature, our study shows a dominant focus for external level integration. This could be attributed to the fact that most of the enterprises nowadays are frequently collaborating with external stakeholders through contract farming, sharing of supply chain information, and company merger. For such situations, enterprises want to integrate their systems and data applications externally with those of the collaborating stakeholders, which could include government organizations, to be able to share data and information. Moreover, this could be explained with the increase in the number of digital technologies, as every enterprise has their own specialized technologies and integrating the technologies externally tends to be the main idea to enhance efficiency in the collaboration. Even though most publications focus on external integration, none of the studies discussed the full spectrum of IS integration, ranging from basic to enterprise-level and even industry-wide integrations.

The third research question focuses on identifying the integration types that can be used to integrate the different technologies within the agri-food ecosystem. Process integration, data, network, and application were identified and examined. Our review shows an emphasis on data integration as a key factor to interoperability. Data being the most addressed integration type could be attributed to both organizational and technical reasons. From an organizational perspective, data and information play a key role in addressing food safety issues, improving traceability and environmental sustainability. The sector is also confronted to deal with large volume and a variety of data as results of the widespread use of IS. Technically, to fully reap the benefits from the data, the sector is therefore required to integrate these data as this often tends to be the main avenue. Although data emerged as the most frequently suggested integration type, in the studies, the discussions were also focused on technical, syntactic, and semantic levels. By validating this outcome using the framework of [147], there seems to be some consistency. However, in the framework of [147], it was seen that IS integration could also be carried out on a higher level such as pragmatic, dynamic, and conceptual levels, which are all mutually supportive. Unfortunately, none of the analyzed studies highlight these higher integrations.

In the reviewed papers, process and network integrations are frequently addressed. This could be because of the role played by process integration in facilitating the alignment of enterprise processes with digital technologies, enhancing collaboration within and outside agri-food organizations. Network integration on the other hand has a key role as the main medium through which the communication between the different systems is realized. It serves as the precondition for any type of IS integration, as it provides direction for integrating different hardware and software components. Surprisingly, application integration was least mentioned in the primary studies, likely because most of the studies focused on data integration. A single standalone application can generate vast amounts of data, prompting researchers to prioritize data integration to ensure data flow and interoperability.

Moreover, as stated by [3,148], a more feasible approach is required for the integration of the processes, data, networks, and applications. In answering the fourth research question, the study identified the integration approaches. As a form of inductive content analysis, we categorized the approaches identified from the studies into (1) point-to-point (P2P), (2) enterprise service bus (ESB), (3) hub-and-spoke, (4) cloud-based, and (5) semantic web integration. This outcome demonstrates that the approaches could be used separately; however, depending on the need and complexity, they can also be combined into hybrid approaches. These findings are in accordance with the conclusion of [146] and results reported by [149] where point-to-point, enterprise service bus, and hub-and-spoke were reported as integration approaches. Contrary to the study by [149], no detailed evidence was found on the underlying architectural patterns for the approaches. While Age of Information (AoI) contributes to the timeliness and freshness of information to minimize delays in communication among systems, this approach was not extensively discussed in the agri-food studies. The absence of a comprehensive discussion can be attributed to the concept’s infancy or the lack of studies specifically addressing Age of Information (AoI) in agriculture. This could be an obstacle to its widespread recognition and practical application. As smart farming advances, AoI is likely to gain more attention in the coming years.

Regarding the most frequently mentioned integration approaches, we discovered that point-to-point (P2P) and cloud-based were common methods employed for integrating IS within the agri-food industry. The reliance on these approaches suggests that both point-to-point (P2P) and cloud-based integration can facilitate the integration of existing legacy systems and recent advanced applications to enhance data-driven decisions.

In terms of their application, point-to-point is useful if the goal for the integration is to enable real-time flow of data between the technologies. In studies demonstrating this approach, it is often used to embed open-source features like weather data, field maps, or pre-built functionalities from third-party applications in enterprise systems. The “spaghetti” architecture of the point–point approach often makes it difficult to scale and enforce integration standards. Cloud-based methods were identified among recent studies, demonstrating their prominence in integrating smart farming cloud solutions. Integrating IS using this approach could empower agri-food stakeholders, especially those in developing countries, to access relevant data and applications from anywhere. This is particularly beneficial in rural areas where agribusinesses are dispersed.

Hub-and-spoke and ESB offer methods for IS integration using message brokers. With hub-and-spoke, because all the applications rely on a single message-oriented middleware, any failure in the central hub affects the connected applications. Enterprise service bus has some qualities to address failures, but like the hub-and-spoke approach, a failure in the bus can also cause a breakdown among participating applications.

In contrast with the other approaches, semantic web integration methods were identified from most recent studies. This approach is recommended for data integration, but due to its underlying approaches (RDF and OWL) and limited application in the agri-food sector, it is often perceived as complex. Due to this, one study proposed that using semantic technologies for data integration requires an expert knowledge in semantic technologies. This may explain why it is not widely adopted in the sector compared to hub-and-spoke. Looking at the emphasis on data integration and rapid evolvement of the technologies in the sector, it can be predicted that more semantic web integration methods will be applied in the future.

The final research question addresses the issues and barriers limiting IS integration in the agri-food sector. Overall, 27 sets of challenges to IS integration were identified from 46 of the primary studies. This result shows that digital technology integration challenges persist in the literature and practice, as some of the issues identified are based on recent research, e.g., 2024 publications. The reported challenges were examined and then grouped into organizational, technological, and data governance.

Technological and data governance issues were identified as the main challenges of IS integration. This finding implies that stakeholders such as system integrators and technology providers might struggle to integrate the different IS across the agri-food sector if both technological and data governance issues persist. Consequently, this could potentially delay the complete integration of IS. The frequent occurrences of technological challenges could be attributed to the fact that most of the integrations occur among software and hardware components, which often come from different developers and technology providers. The issues pertaining to data governance include data security, privacy, and ethical and legal challenges. These were observed mainly among papers published in recent years. This is likely due to the increasing regulatory and compliance demands in food supply chains. Although cyber security systems were acknowledged in the existing technologies, the reviewed papers failed to address data governance issues related to cyber security threats, including system vulnerabilities and ransomware attacks, which could compromise integrated IS and the broader food supply chain. This gap limits the understanding of how cyber security risks could be mitigated. This limitation could be due to the minimal focus on cyber security system results derived from the literature studies.

Addressing these two primary challenges is therefore essential for a seamless integration of IS, which could potentially transform the agricultural sector towards increased efficiency and sustainability. In order to address the technological challenges, it was found that semantic web approaches, microservices, and SOA could be utilized. A semantic web integration approach can help ensure that data from various IS are meaningfully exchanged and understood. Likewise, we identified technologies such as blockchain and the adoption of multi-instance architecture design as possible solutions to address data governance challenges.

Besides the technological and data governance challenges, the results indicated that obstacles to integration could come from organizational issues such as the lack of clarity of organizations’ business models and inadequate skilled resources. However, no evidence was found among the existing studies discussing the interplay between the main challenges and organizational issues. Such results would have been revealing, as they would shed light on the impact of socio-technical perspectives, for instance, in global supply chains where data needs to be shared between developed and developing regions, as well as between large and small organizations. Studies such as [150] contributed to addressing power asymmetries and cultural resistance to data sharing. This SLR outcome can be further explored in future research that incorporates theories such as Actor–Network Theory, which was demonstrated in existing studies [151,152]. 

## 5. Conclusions

The integration of information systems (IS) into fully connected systems is widely addressed as a fundamental challenge for smart farming. However, realizing such a full smart farming integration is a multi-faceted issue that encompasses not only the specific application of the individual IS but also other crucial aspects of integration.

This study conducted a comprehensive review of the different dimensions of integration of smart information systems in the agri-food sector. By using a systematic literature review approach, 74 scientific articles were analyzed concerning (1) the various IS addressed including emerging technologies, (2) the level of integration, (3) the various ways in which IS could be integrated, (4) the approach for integrating IS, and (5) the challenges associated with integrating IS and corresponding possible solutions for mitigating the challenges.

By providing an overview from a broader perspective into these dimensions of IS integration, the study delivers the state-of-the-art into the reciprocal relationship between the agri-food IS and how these technologies can be appropriately integrated. By doing so, the study provides structured information and understanding for agri-food producers, retailers, and technology providers. These insights will help facilitate the integration of the fragmented and siloed IS landscape, leading to data-driven smart farming that increases food production, mitigates climate change, and optimizes resource usage. We also believe that the solutions proposed for the challenges to integration could be useful for practitioners to mitigate the risks associated with integrating existing and new technologies.

Despite this, we observed some gaps in the literature for which further research is needed. None of the studies highlight organizational and digital maturity levels in relation to digital technology integration. Based on the review results, especially the integration challenges, our future research will focus on assessing smart farming maturity using capability maturity models. The review acknowledges AI as a suitable approach for enabling IS integration. This was not discussed in the current studies. In our future research, we will explore in detail the potential role of AI in IS integration and propose new integration approaches. This will help utilize AI capabilities to harmonize data from disparate smart farming technologies. Finally, while integration issues concerning data governance were prioritized in the literature, nonetheless, most of the studies tended to focus on integration types, approaches, and challenges with less attention paid to the governance and socio-technical linkages of digital technology integration. In-depth follow-up research on data governance issues especially cyber security risks in IS integration and frameworks for addressing such threats is also needed to investigate this important topic area.

## Figures and Tables

**Table 1:** List of inclusion criteria (IC).

No.	Selection Criteria
IC 1	Articles published in 2010 to 2024
IC 2	Full text of publication must be available
IC 3	Article must be in English
IC 4	Publication must not be duplicated or found in other databases
IC 5	Publication must highlight information system integration
IC 6	Article must discuss information systems for agri-food sector
IC 7	Publication must be a survey paper

**Table 2:** List of quality assessment criteria.

No.	Question	1 = Yes	0.5 = Partial	0 = No
Q1	Study aims clearly stated			
Q2	Study scope and context defined clearly			
Q3	Study materials and methods documented clearly			
Q4	All research questions answered			
Q5	Study’s main findings reported clearly			
Q6	Conclusions stated clearly and relate to the aim of the study			

**Table 3:** Information systems in the agri-food sector addressed in the studies.

Categorization of Information Systems	Specific Technology
Data processing and analytics systems	Machine learning, AI, big data analytics, data mining, blockchain, data spaces, robotics, semantic technologies, ontology systems, and decision support systems.
Business information systems	Farm management information system (FMIS), enterprise resource planning (ERP), supply chain systems, mobile (smartphone) applications, market information systems, production, quality management systems, and transport management applications.
IoT and sensor systems	IoT, sensors, UAVs (e.g., drones), monitoring cameras, RFID, electronic tags, satellites, code scanning guns, GIS, and weather stations.
Other systems	Cyber security, digital twin, and cloud computing.

## Abbreviations

The following abbreviations are used in this manuscript:

AGROVOC	Agriculture vocabulary
API	Application programming interface
AI	Artificial intelligence
AoI	Age of Information
DSL	Domain-specific language
ERP	Enterprise resource planning
ESB	Enterprise service bus
FMIS	Farm management information systems
GIS	Geographic information systems
GPRS	General packet radio service
IC	Inclusion criteria
IS	Information systems
IoT	Internet of Things
ML	Machine Learning
OWL	Web Ontology Language
P2P	Point-to-point
RQ	Research question
RFID	Radio frequency identification
RDF	Resource Description Framework
SLR	Systematic literature review
SOA	Service-oriented architecture
SoS	System of systems
UAVs	Unmanned aerial vehicles

## Appendix A

**Table A1:** Data extraction form.

ID	Question
	**Section A: General Information**
Q1.	ID
Q2.	Title
Q3.	Repository
Q4.	Year
Q5.	Authors
Q6.	SLR Category
Q7.	Date of data extraction
	**Section B: Description**
Q8.	Study domain
Q9.	What IS/ software are currently used as reported in the paper
Q10.	For each IS/ software mentioned in Q8 indicate the key feature or main function
Q11.	Identified level of integration of IS
Q12.	Identified type of integration of IS
Q13.	Identified approach for IS integration
Q14.	Identified challenges for IS integration
Q15.	Provided solution for the challenges mentioned in Q13 if mentioned
	**Section C: Study evaluation**
Q16.	Personal remark
Q17.	Quality assessment

## Appendix B

**Table A2:** The selected 74 studies.

Study ID	Authors	Title
P01	Allemang D. (2019)	Sustainability in data and food
P02	Almadani B., Mostafa S.M. (2021)	IIoT based multimodal communication model for agriculture and agro-industries
P03	Alreshidi E. (2019)	Smart Sustainable Agriculture (SSA) solution underpinned by Internet of Things (IoT) and Artificial Intelligence (AI)
P04	Amiri-Zarandi M. et al. (2022)	A Platform Approach to Smart Farm Information Processing
P05	Bazzi C.L. et al. (2019)	AgDataBox API–Integration of data and software in precision agriculture
P06	Bhat S.A. et al. A. et al. (2022)	Agriculture-Food Supply Chain Management Based on Blockchain and IoT: A Narrative on Enterprise Blockchain Interoperability
P07	Blank S. et al. (2013)	IGreen: A ubiquitous dynamic network to enable manufacturer independent data exchange in future precision farming
P08	Branco F. et al. (2021)	An integrated information systems architecture for the agri-food industry
P09	Brewster C. et al. (2017)	IoT in Agriculture: Designing a Europe-Wide Large-Scale Pilot
P10	Budaev D. et al. (2018)	Conceptual design of smart farming solution for precise agriculture
P11	Chen N. et al. (2015)	Integrated open geospatial web service enabled cyber-physical information infrastructure for precision agriculture monitoring
P12	Delgado J.A. et al. A. et al. (2019)	Big Data Analysis for Sustainable Agriculture on a Geospatial Cloud Framework
P13	Deng M. et al. (2013)	Web-service-based monitoring and analysis of global agricultural drought
P14	Devare M. et al. (2021)	AgroFIMS: A Tool to Enable Digital Collection of Standards-Compliant FAIR Data
P15	Durrant A. et al. (2021)	How might technology rise to the challenge of data sharing in agri-food?
P16	Fang W. et al. (2013)	Study for efficient integration and sharing architecture for agriculture data resources
P17	Fernandes M.A. et al. A. et al. (2013)	A framework for wireless sensor networks management for precision viticulture and agriculture based on IEEE 1451 standard
P18	Ferrández-Pastor F.-J. et al. (2022)	Agricultural traceability model based on IoT and Blockchain: Application in industrial hemp production
P19	Fountas S. et al. (2015)	Farm machinery management information system
P20	Gallinucci E. et al. (2020)	Mo.Re.Farming: A hybrid architecture for tactical and strategic precision agriculture
P21	Giroux S.A. et al. A. et al. (2019)	A high-frequency mobile phone data collection approach for research in social-environmental systems: Applications in climate variability and food security in sub-Saharan Africa
P22	Hsu T.-C. et al. C. et al. (2020)	A Creative IoT agriculture platform for cloud fog computing
P23	Janssen S.J.C. et al. C. et al. (2017)	Towards a new generation of agricultural system data, models, and knowledge products: Information and communication technology
P24	Junior C.H. et al. (2019)	The adoption stages (Evaluation, Adoption, and Routinisation) of ERP systems with business analytics functionality in the context of farms
P25	Khan F.A. et al. A. et al. (2019)	Cotton crop cultivation oriented semantic framework based on IoT smart farming application
P26	Khatoon P.S., and Ahmed M. (2022)	Importance of semantic interoperability in smart agriculture systems
P27	Kour V.P., and Arora S. (2020)	Recent Developments of the Internet of Things in Agriculture: A Survey
P28	Kruize J.W. et al. (2016)	A reference architecture for Farm Software Ecosystems
P29	Lezoche M. et al. (2020)	Agri-food 4.0: A survey of the Supply Chains and Technologies for the Future Agriculture
P30	Morais R. et al. (2019)	mySense: A comprehensive data management environment to improve precision agriculture practices
P31	Munz J. et al. (2020)	Exploring the characteristics and utilisation of Farm Management Information Systems (FMIS) in Germany
P32	Ngo V.M., and Kechadi M.-T. (2021)	Electronic farming records–A framework for normalising agronomic knowledge discovery
P33	O’Grady M. et al. (2021)	Service design for climate-smart agriculture
P34	Pang Z. et al. (2015)	Value-centric design of the internet-of-things solution for food supply chain: Value creation, sensor portfolio and information fusion
P35	Ram C.R.S. et al. (2020)	Internet of Green Things with autonomous wireless wheel robots against green houses and farms
P36	Rejeb A. et al. (2019)	Leveraging the Internet of Things and blockchain technology in Supply Chain Management
P37	Reza A.W. et al. (2022)	Smart Pre-Seeding Decision Support System for Agriculture
P38	Roy S.K., De D. (2022)	Genetic Algorithm based Internet of Precision Agricultural Things (IopaT) for Agriculture 4.0
P39	Santana C. et al. (2021)	Increasing the availability of IoT applications with reactive microservices
P40	Si H.-P. et al. (2013)	Method for agriculture data integration and sharing based on SOA
P41	Singh S. et al. (2020)	A framework for successful IoT adoption in agriculture sector: A total interpretive structural modelling approach
P42	Sivamani S. et al. (2013)	A smart service model based on ubiquitous sensor networks using vertical farm ontology
P43	Taylor K., Amidy M. (2020)	Data-driven agriculture for rural smallholdings
P44	Teucher M. et al. (2022)	Digital In Situ Data Collection in Earth Observation, Monitoring and Agriculture—Progress towards Digital Agriculture
P45	Tóth K., Kučas A. (2016)	Spatial information in European agricultural data management. Requirements and interoperability supported by a domain model
P46	Trilles S. et al. (2020)	Development of an open sensorized platform in a smart agriculture context: A vineyard support system for monitoring mildew disease
P47	Tzounis A. et al. (2017)	Internet of Things in agriculture, recent advances, and future challenges
P48	van Evert F.K. et al. (2017)	Big Data for weed control and crop protection
P49	Verdouw C. et al. (2019)	Architecture framework of IoT-based food and farm systems: A multiple case study
P50	Verdouw C.N. et al. (2014)	Towards a Smarter Greenport: Public-Private Partnership to Boost Digital Standardisation and Innovation in the Dutch Horticulture
P51	Wang J. et al. (2020)	Data communication mechanism for greenhouse environment monitoring and control: An agent-based IoT system
P52	Yue P. et al. (2014)	Google fusion tables for managing soil moisture sensor observations
P53	Zhang X. et al. (2020)	Blockchain-based safety management system for the grain supply chain
P54	Aydin S., Aydin M.N. (2020)	Semantic and syntactic interoperability for agricultural open-data platforms in the context of IoT using crop-specific trait ontologies
P55	Schuster E.W. et al. (2011)	Machine-to-machine communication for agricultural systems: An XML-based auxiliary language to enhance semantic interoperability
P56	Kim J.Y. et al. (2015)	Open farm information system data-exchange platform for interaction with agricultural information systems
P57	Aitlmoudden O. et al. (2023)	A Microservices-based Framework for Scalable Data Analysis in Agriculture with IoT Integration
P58	Falcão R. et al. (2023)	A Reference Architecture for Enabling Interoperability and Data Sovereignty in the Agricultural Data Space
P59	Gebresenbet G. et al. (2023)	A concept for application of integrated digital technologies to enhance future smart agricultural systems
P60	Khatoon P.S. et al. (2023)	Design and development of dynamic Agri-ontology for IoT interoperability
P61	Lou J.-T. et al. (2023)	Blockchain-based privacy-preserving data-sharing framework using proxy re-encryption scheme and interplanetary file system
P62	Morales-García J. et al. (2023)	SEPARATE: A tightly coupled, seamless IoT infrastructure for deploying AI algorithms in smart agriculture environments
P63	Moysiadis T. et al. (2023)	AgriFood supply chain traceability: data sharing in a farm-to-fork case
P64	Roussaki I. et al. (2023)	Building an interoperable space for smart agriculture
P65	San Emeterio de la Parte M. et al. (2023)	Big Data and precision agriculture: a novel spatio-temporal semantic IoT data management framework for improved interoperability
P66	Abhilash Sam Paulstin K.C. et al. C. et al. (2024)	Cloud-Based Top-Down and Bottom-Up Approach for Agriculture Data Integration
P67	Barriga A.; Barriga J.A. et al. A. et al. (2024)	Model-Driven Development Towards Distributed Intelligent Systems
P68	Brewster, C. et al. (2024)	Data sharing in agricultural supply chains: Using semantics to enable sustainable food systems
P69	Jing R. et al. (2024)	Knowledge graph for integration and quality traceability of agricultural product information
P70	Kalimuthu V.K. et al. (2024)	Blockchain Based Secure Data Sharing in Precision Agriculture: a Comprehensive Methodology Incorporating Deep learning and Hybrid Encryption Model
P71	Romera A.J. et al. (2024)	Digitalization in agriculture. Towards an integrative approach
P72	Tahir, HA. et al. A. et al. (2024)	AgriChainSync: A Scalable and Secure Blockchain-Enabled Framework for IoT-Driven Precision Agriculture
P73	Urdu D. et al. (2024)	Aligning interoperability architectures for digital agri-food platforms
P74	Granell C. et al. (2024)	Conceptual architecture and service-oriented implementation of a regional geoportal for rice monitoring

## Appendix C

**Table A3:** The main types of IS integration.

Main types of Integration	No. of Papers	Study
Process	23	[27,28,48,71,72,80,83,86,88,97,102,105,110,112,113,116,117,118,122,131,132,153]
Application	10	[27,28,48,71,80,83,97,116,117,118]
Data	69	[3,18,25,27,28,29,48,68,69,70,71,72,73,74,75,76,77,78,79,80,81,82,83,84,85,86,88,94,95,96,97,98,99,100,101,102,103,104,105,106,107,108,109,110,111,112,113,114,115,116,117,118,120,121,122,123,124,125,126,127,128,129,130,131,132,153,154,155,156,157,158]
Network	16	[28,48,71,72,80,83,86,92,98,110,112,113,117,118,122]

## Appendix D

**Table A4:** Approaches for integrating systems in agri-food sector.

Approaches of Integration	No. of Papers	Study
Point-to-point (P2P)	17	[25,28,48,71,76,78,86,97,100,102,105,114,116,118,122,124,132]
Enterprise service bus (ESB)	10	[76,80,97,98,106,110,111,112,125,131]
Cloud-based integration	14	[3,18,69,72,74,77,79,103,109,113,123,128,130,153]
Hub-and-spoke	6	[68,78,86,94,99,104]
Semantic web integration	10	[27,68,73,75,79,84,95,107,114,154]
Others	10	[16,29,82,84,87,101,108,120,121,127]
